# Can investments in manure technology reduce nutrient leakage to the Baltic Sea?

**DOI:** 10.1007/s13280-019-01251-5

**Published:** 2019-10-03

**Authors:** Torbjörn Jansson, Hans Estrup Andersen, Berit Hasler, Lisa Höglind, Bo G. Gustafsson

**Affiliations:** 1 Department of Economics, Swedish University of Agricultural Sciences and AgriFood Economics Centre, P.O. Box 7013, 750 07 Uppsala, Sweden; 2 Department of Bioscience, Aarhus University, Vejlsøvej 25, 8600 Silkeborg, Denmark; 3 Department of Environmental Science, Aarhus University, Frederiksborgvej 399, 4000 Roskilde, Denmark; 4 Stockholm University Baltic Sea Centre, 106 91 Stockholm, Sweden; 5 Tvärminne Zoological Station, University of Helsinki, J.A. Palménin tie 260, Hanko, Finland

**Keywords:** Baltic Sea, Common agricultural policy, Eutrophication, Modelling, Nitrogen

## Abstract

In this study, quantitative models of the agricultural sector and nutrient transport and cycling are used to analyse the impacts in the Baltic Sea of replacing the current Greening measures of the EU’s Common Agricultural Policy with a package of investments in manure handling. The investments aim at improving nutrient utilization and reducing nitrogen leaching, based on the assumption that lagging farms and regions can catch up with observed good practice. Our results indicate that such investments could reduce nitrogen surpluses in agriculture by 18% and nitrogen concentrations in the Baltic Sea by 1 to 9% depending on the basin. The Greening measures, in contrast, are found to actually increase nitrogen leaching.

**Electronic supplementary material:**

The online version of this article (10.1007/s13280-019-01251-5) contains supplementary material, which is available to authorized users.

## Introduction

Eutrophication has been a major problem in the Baltic Sea for decades and continues be so (HELCOM 2018; Reusch et al. 2018), and during the twentieth Century, dead bottoms expanded over significant areas and excessive cyanobacteria blooms occur frequently (e.g. Gustafsson et al. 2012).

Agriculture is the main cause of nutrient loads to the Baltic Sea (Reusch et al. 2018), and the relative importance of the agricultural loads has increased as loads from wastewater treatment have decreased.

In a European context, the Baltic Sea is important for several reasons; the drainage basin covers a large part of the land area in the EU (17%) and EU member states make up 75.4% of the drainage basin of the Baltic Sea. Nutrient loads to the Baltic Sea are directly and indirectly influenced by EU policies in several areas: the implementation of EU directives such as the Water Framework Directive (WFD), The Nitrates Directive and the Marine Strategy Framework Directive (MSFD), as well as the EU Common Agricultural Policy (the CAP).

Animal husbandry and the resulting nutrient losses from manure are regulated especially by the Nitrates Directive, which has facilitated strict regulations of manure by setting compulsory requirements for manure handling (storage requirements to prevent application on frozen soil), as well as maximum allowable Livestock Units per hectare. Requirements differ between countries around the Baltic. For instance, the storage capacity requirement for manure ranges from 12 months in Finland to 6 months in Poland and Latvia (ECA 2016). Similarly, the implementation of the WFD has also resulted in implementation of measures to reduce nutrient loads, in many countries closely linked to the agri-environment-climate measures (AECMs) and subsidy schemes implemented as part of the Rural Development Programme of the CAP. Among other requirements, the CAP includes three compulsory greening requirements, with the objective to “deliver environmental and climate benefits” (European Commission 2011). Greening was introduced in the 2013 CAP reform, and Member States must allocate 30 per cent of their national CAP Pillar 1 budget ceilings for the associated annual payments (Hart 2015). None of the greening requirements is directly linked to the environmental effects (performance) but to measures anticipated to provide a number of positive effects; including nutrient abatement.

Former studies (Westhoek et al. 2012, Alliance Environment and Thuenen Institute 2017; ECA 2017; Gocht et al. 2017) suggest that the broad greening measures are not very effective from an environmental perspective. In contrast to the more general greening requirements, more targeted measures, like manure management requirements, have proven to be effective in improving nutrient utilization efficiency, and to decrease nutrient loads to the marine environment (Windolf et al. 2016; Hong et al. 2017; McCrackin et al. 2018). Requirements and financial support for improving the manure management in the Baltic Sea region might therefore be more effective, targeted methods than greening when it comes to nutrient abatement.

This study investigates the effects of removing the greening requirement and implementing alternative technological measures for nitrogen abatement in agriculture. Focus is on measures directed towards the handling of manure on farm level with the aim to reduce nutrient surpluses and loads into the Baltic Sea. The scenarios addressed include removal of the greening requirements and the introduction of a package of investments in technology for improved storage and utilization of manure.

To enable quantitative analysis of the scenarios, we use a set of three simulation models, described in the following section. In that section, we also describe the scenario set-up, the specific modelling of the nutrient balance for agriculture in detail, and document the modifications to the standard model that were introduced for the purpose of this analysis. Then, we present the results with specific focus on nutrient utilization efficiency, leaching, loads to the marine environment as well as the effects in the Baltic Sea. The final section concludes the paper with a discussion.

## Materials and Methods

### A chain of specialized models

**Fig. 1:**
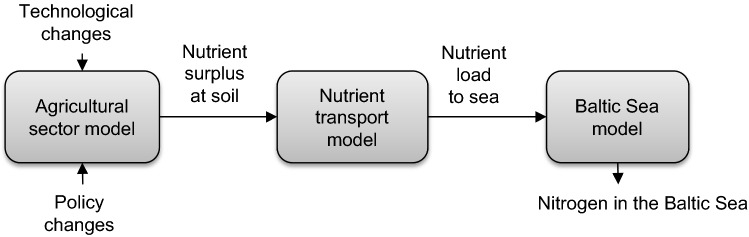
Model chain translating changes in policy and technology to impacts on nitrogen in the Baltic Sea

We utilize a chain of three specialized models as illustrated in Fig. 1. Impacts of technological and policy changes on agriculture is the starting point, obtaining as a result the impacts on production, land use and, not least important, the balance of nutrient inputs and removals from the soils. The nutrient surplus is fed into a hydrological flow model that computes the riverine loads to the Baltic Sea. Finally, a marine ecosystem model of the Baltic Sea is used to compute impacts on nitrogen concentrations in the various basins of the Baltic Sea.

### The agricultural sector model CAPRI

CAPRI is a partial equilibrium model for the agricultural sector of the European Union and global trade in food and agricultural commodities. The model has been used and documented in many applied analyses (e.g. Himics et al. 2018; Pérez-Domínguez et al. 2016; Renwick et al. 2013).

Agricultural production is modelled in 276 regional farm models: one farm model for each NUTS2 region in the EU, Norway, Western Balkans and Turkey. The model covers 51 agricultural commodities. These are produced by 50 crop and animal activities in each of the regions, using 9 general inputs, 3 crop-specific inputs, 6 intermediate crop outputs, 12 intermediate animal outputs (including manure), 3 types of mineral fertiliser (N, P, K) and 10 tradable and non-tradable feed inputs. Each regional farm model optimises regional agricultural income at given prices and subsidies and is constrained by land availability, policy variables and feed and plant nutrient requirements in each region. CAPRI computes nutrient balances on the level of NUTS2 for each group of crops and each of the three nutrients N, P and K, as described in greater detail in Electronic Supplementary Material 1. Market equilibria for prices and demand are computed in a global trade model.

The results of a simulation on NUTS2 level can be downscaled to a finer spatial resolution called *Homogeneous Spatial Mapping Units* (HSMU) (Kempen 2013). In this paper, we use such downscaled results as inputs to the agro-hydrological nutrient transport model, working at the level of catchments, described in the following section. Each HSMU consists of clusters of 1 km grid cells that are similar in terms of soil type, climate, slope, elevation and NUTS3 affiliation. HSMU are generally discontinuous, and vary in size depending on the diversity of the underlying area. There are about 175 000 HSMU in EU28, and of those 34 882 (average size: 3630 ha) are found in the Baltic Sea drainage area (see Fig. 3 for a visualization), giving an average of 298 HSMU per catchment for the 117 catchments modelled in this paper. The disaggregation uses a priori distributions of crops obtained from estimations based primarily on (i) satellite imagery data of the CORINE land cover data base, (ii) in situ observations of land use (36 crop land, 2 permanent grassland classes) of the LUCAS dataset, and (iii) CAPRI regional agricultural production data in the relevant ex-post period. The downscaled model results can be used to calculate also the nutrient balances of each HSMU following a simulation with the model.

The supply model contains a rich set of agricultural policy instruments: There are subsidies based on areas or animal herds such as the Basic Payment Scheme (BPS, decoupled payments), Voluntary Coupled Support (coupled payments), special aid to young farmers and smaller farms, and the national Nordic Aid in Sweden and Finland (coupled subsidies to selected production activities). Of particular interest for us here are the Greening restrictions, which aim to enhance the environmental impacts of the CAP, and that requires the farmers/regions to (i) set aside a share of land as “ecological focus area”, (ii) maintain a minimum number of crops, “crop diversity”, and (iii) not reduce the share of land that is grassland. The greening restrictions are compulsory, and 30% of the BPS amount is conditional on complying with the greening measures. The model also implements selected policies of the second pillar of the CAP, most importantly the agri-environmental schemes that include subsidies to grasslands in many regions, and support to areas with natural constraints.

### The agro-hydrological nutrient transport model

Agricultural management practices are among the major drivers of agricultural nutrient losses. In a previous study (Andersen et al. 2016), an agro-hydrological N-transport model for the Baltic Sea drainage basin was developed which runs at a high spatial resolution and is computationally effective. The model was developed from a dataset of more than 4000 agricultural fields with combinations of climate, soils and agricultural management, which overall describe the variations found in the Baltic Sea drainage basin. The soil–vegetation–atmosphere model Daisy (Hansen et al. 1991) was used to simulate N loss from the rootzone of all agricultural fields in the dataset. From the dataset of Daisy simulations, the most important drivers for N loss were identified by multiple regression analysis and formed into a statistical N loss model. In the present study, Andersen et al. (2016)’s statistical model of N losses is applied at the HSMU scale driven by the following inputs provided partly by CAPRI: crop type, farm type, total N input to the crop including fertilizer, manure, N fixation, atmospheric N deposition, and N in the seed, and additionally information on clay content and soil carbon content in the topsoil.

Nitrogen leached from the rootzone of agricultural fields and from other areas is subjected to denitrification, often referred to as N retention, during transport to the sea through groundwater and surface waters (streams, lakes and wetlands). Andersen et al. (2016) combined own work with the work of Stålnacke et al. (2015) into estimates of, respectively, groundwater and surface water N retention in the entire Baltic Sea drainage basin subdivided into 117 individual catchments. For each catchment, the resulting N loading to the Baltic Sea can be calculated by combining N losses at the HSMU scale with catchment-wide N-retention estimates.

### The Baltic Sea model BALTSEM

The Baltic Sea is a huge estuary with significant horizontal and vertical salinity gradients. The coupled physical–biogeochemical model BALTSEM is developed to simulate the spatiotemporal effects of nutrient inputs and physical drivers on the status of the marine environment. The model features mechanistic process descriptions for water circulation and mixing, and biogeochemical cycling of the major nutrients (N, P and Si) in water column and sediments. Details of the model construction is available in Gustafsson et al. (2012), Savchuk et al. (2012) and Gustafsson et al. (2017). The model has been used for management purposes in determining Maximum Allowable Inputs used by HELCOM (HELCOM 2013a, b).

### Scenarios

We analyse the effects of replacing the current Greening requirements, which previous studies have shown to be inefficient in terms of environmental benefits, with a selection of investments in manure storage and handling technologies. In order to isolate the effects of changes in policies (removing greening) and technology, two scenarios were simulated and compared to a *Reference* scenario implying no change (see Table 1 for an overview).

**Table 1:** Scenarios analysed and the associated changes to policies and technologies

Scenario name	CAP payments	Manure storage	Fertilization technology	Manure composition
Reference	Current policies continued up to 2030	As observed 2017	As observed 2017	As observed 2017
No greening	Greening restrictions and payments removed	As “reference”	As “reference”	As “reference”
Manure investments	Greening restrictions and payments removed	All manure is stored in facilities with > 9 m capacity	All liquid manure is spread using hoses and injection	Share of liquid increased to ≥ 0.75

The *Reference* scenario represents the current policies continued up to 2030 and thus includes the three greening requirements as well as the BPS and the various coupled subsidies mentioned in the CAPRI model description. Manure storage and handling in *Reference* are based on estimates of current practices, implying considerable differences in nutrient utilization across countries and regions. The data and technical assumptions are described more closely in the following section.

In the *No greening* scenario, manure storage and technology are kept fixed as in *Reference*, while the three greening measures (ecological focus area, grassland maintenance and crop diversification) are removed and CAP budget is reduced by 30% corresponding to the greening payments. Removing the greening not only releases budget, but also allows grassland to be abandoned if unprofitable and set aside to be reduced.

The main technology scenario, *Manure investments* builds on *No greening* and additionally assumes that it is possible to use some of the released budget to invest and improve manure handling technologies on farms so that a certain minimum standard is achieved everywhere. We focus on three complementary technological measures: (a) Increased manure storage capacity to at least 9 months of production, (b) replacing the use of broad-spread techniques for the application of liquid manure with a combination of hoses and injection technology (50% share of each), and (c) modernizing facilities to phase out solid manure systems, so that the share of liquid manure is at least 75%. *Manure investments* implies a technological catching-up to good practice in all NUTS2-regions. Note that we do not model the decisions of the farmers to adopt the new technology, but simply assume that it is possible to achieve with the budget released from the removal of greening (30% of pillar 1, or 12 billion euro per annum for EU28) or with regulation. Modelling technological adoption is beyond the scope of the current paper.

### Modelling the Fertilizer Value of manure

The investments in manure storage capacity and the uptake of application technology are not endogenous decisions in the simulation model, but exogenously computed before simulation as part of the scenario definition. This is an extension of the standard CAPRI model. The key assumptions of the model linking storage capacity to manure efficiency (fertilizer value, FV, relative to mineral fertilizer) are the following:

- The FV depends on timing of application, technology of application, and the shares of solid/liquid manure as defined in Eq. 1.
- The timing of application depends on storage capacity as defined in Eq. 2.
- The shares of solid/liquid manure are exogenous data
- The shares of technologies (broad spread, hoses, injection) are exogenous data

We compute the average fertilizer value in region $r$, ${\text{FV}}_{r}$, as the share-weighted mean of the fertilizer value ${\text{FV}}_{\text{kit}}$ of manure when coming from different systems *k *= {liquid, solid}, applied with different technologies *i* = {broad, hoses, injection}, and with different timings $t = \left\{ {{\text{spring}},{\text{summer/fall}},{\text{winter}}} \right\}$.

Definition of fertilizer value in each region

1 $$ {\text{FV}}_{r} = \mathop \sum \limits_{kit}   {\text{FV}}_{kit} a_{rk} b_{rki} c_{rt} $$

where $a_{rk}$ is the share of manure coming from liquid/solid systems (*k*), $b_{rki}$ is the share of manure applied using technology *i,* where the advanced options “hoses” and “injection” are not available for solid manure systems, and $c_{rt}$ is the share of manure applied in time period *t.*

For the timing of application, we assume a linear relation to the storage capacity defined as in Eq. 2:

Timing of application as a function of storage capacity

2 $$ c_{rt} = \beta_{t} x_{r} + \alpha_{t} $$

On the left-hand side, $c_{rt}$ shows the share of liquid manure that is applied in each of the three time periods $t = \left\{ {{\text{spring}},{\text{summer/fall}},{\text{winter}}} \right\}$, in each region $r$, depending on the regional average storage capacity $x_{r}$ and the two estimated parameters $\alpha_{t}$ and $\beta_{t}$. Storage capacity is defined as the share of liquid manure that is kept in storage facilities with at least 9 months of capacity, as this is the minimum capacity allowing optimal timing of application of manure. The linear function is such that $\mathop \sum \limits_{t} c_{rt} = 1$ for any $x_{r}$.

In order to implement the two equations above, we need estimates for the parameters $FV_{kit}$, $\alpha_{t}$ and $\beta_{t}$, data on the technology shares $a_{rk}$ and $b_{rki}$, and data on storage capacity $x_{r}$. This is the topic for the next section. Electronic Supplementary Material 2 shows the computation of the resulting FV for the eight countries around the Baltic Sea.

### Data and parameters

Standard values of fertilizer value as a function of timing of manure application and spreading technology were provided by Landskontoret for Planteavl (1989) and Landskontoret for Planteavl (2006) and aggregated across manure categories to arrive at a level of aggregation suitable for the CAPRI model. The parameters are shown in Table 2. Solid manure is only applicable using broad-spread technology. For liquid manure, springtime application is the most efficient, in particular if done with injection technology. This drives the results later on, as increased storage capacity increases the share of spring-time application.

**Table 2:** Fertilizer value $FV_{\text{kit}}$ for types of manure, technologies of application and timing of application (percent, relative to mineral fertilizer)

Manure type (k)	Application technology (i)	Timing of application (t)		
		Spring	Summer/fall	Winter
Solid	broad spr.	40	25	35
Solid	Injection	n.a.	n.a.	n.a.
Solid	Hoses	n.a.	n.a.	n.a.
Liquid	broad spr.	45	25	30
Liquid	Injection	65	60	30
Liquid	Hoses	55	45	30

Data on the number of animals kept in liquid and solid systems for eight types of animals in all EU countries^1^ were incorporated into CAPRI in a previous project, called MITERRA-EUROPE, as described in Velthof et al. (2009). These data are used to compute the shares $a_{rk}$ in standard CAPRI and are used also in the present study.

For the application technology shares $b_{ri}$, we use the results of a comprehensive farm survey covering Estonia, Poland, Denmark, Sweden and Finland (Hasler et al., 2018). Where such results are not available, we use information from Bioteau et al. (2009) as a fall-back position.

The computation of application timing in Eq. 2 requires data on the share $x_{r}$ of manure that is stored in facilities with greater storage capacity than 9 months. This share is rarely available. Instead, we have access to various surveys of the average storage capacity in number of months. In order to make use of the estimated coefficients in Eq. 2, we calibrated a linear transformation function from storage capacity to relative storage capacity in the following way: For Denmark in 1998, average storage capacity was 9 months, and the share of manure stored in facilities with at least 9 months of capacity was 90%. Assuming that 0 month’s average storage capacity gives zero percent storage in facilities with at least 9 months capacity, we obtain two points on a presumed linear function. Thus, we assume that $x_{r} = \hbox{min} \left\{ {1,0.10m_{r} } \right\}$, where $m_{r}$ is the average storage capacity in months in region $r$.

The average storage capacity in number of months was taken from a combination of sources to obtain a complete dataset. The sources are ranked with respect to (subjective) reliability and consistency, giving priority to national statistics (available for Sweden) followed by expert data (for Denmark, Finland and Estonia) followed by the somewhat older data on country level in Pain and Menzi (2003), finally completed, if still missing (which only happens for countries outside of the Baltic Sea drainage basin and thus is of less interest) by data from Bioteau et al. (2009). Albeit the Bioteau et al.’s (2009) dataset is comprehensive, we refrained from using it where other data was available, for methodological reasons: The authors collected survey responses from regional experts for 176 NUTS 2 regions. By organizing regions into groups according to climate and Livestock Production Systems (LPS), they formed a dataset containing clusters of regions with a percentage value for storage capacity and spreading technique,^2^ representing 243 European regions. The clustering was used for a different purpose in Bioteau et al. (2009), and using their data to compute values for the countries used in our study resulted in estimates that were based on just one or two observations and thus the results were deemed as less reliable for our purposes.

We are aware of the fact that most of the data on manure storage and technologies are old, and of different origins for different member states. For instance, the storage capacity for liquid manure in Estonia is 10 months in Pain and Menzi (2003) compared to only 4.3 months in Bioteau et al. (2009). For Denmark, where expert data claim that 90% of manure is stored in facilities with at least 9 months of capacity, Bioteau et al. (2009) report 6 months of capacity on average. For application technologies, we also find large differences. For instance, the recent data of Hasler et al. (2018) show only 0.8% broad-spread technology in Denmark, whereas Bioteau et al. (2009) report 49.4%, and for Estonia, the more recent data report 41.9% use of the best (injection) technology, whereas Bioteau et al. report zero use of injection and 80% broad spread. For Poland, the more recent survey finds larger shares of broad-spread technology (93.5%), whereas the old survey finds lower shares (54–67% across regions). It is certainly possible that structural changes and investments in the sector have changed the average technology during the past decade(s) and will continue to do so up to 2030, but no clear trends can be identified based on the available surveys. There are thus large uncertainties, and an updated and comprehensive survey would be valuable.

The parameters $\alpha$ and $\beta$ of Eq. 2 (defining timing of application depending on storage capacity) were estimated based on Danish data recorded during 1991–1998 (Grant et al. 1999) in which period there was a sharp increase in manure storage capacity and improved timing of manure application due to legal requirements. With the increasing storage capacity, the rate of springtime application increases to a maximum of 90%, whereas the summer application rate drops to 10% and winter application rate drops to zero, reflecting the possibility of farmers to store manure over the winter. Letting *y* be the share (percent) of *N* applied in the period and *x* the share (%) of manure stored in facilities with more than 9 months of storage capacity, the linear functions estimated were as follows: For *spring* time application, *y* = 0.6141*x* + 29.383 (*R*^2^ = 0.9724). For *summer *+* autumn* application, *y* = − 0.4361*x* + 52.902 (*R*^2^ = 0.9587). For *winter* application, *y* = − 0.178*x* + 17.714 (*R*^2^ = 0.6201). Further details of the estimation are shown in Electronic Supplementary Material 3 to this paper.

## Results

**Fig. 2:**
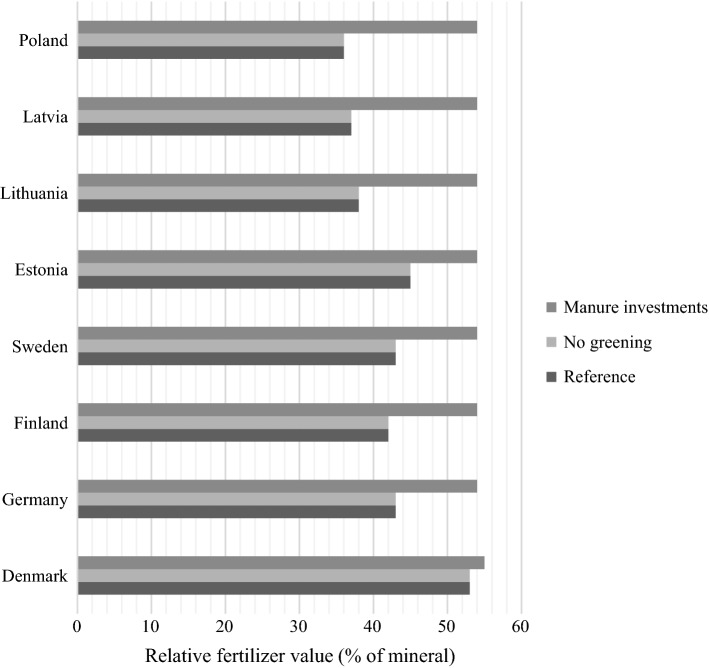
Manure fertilizer value of N in the different scenarios (relative to mineral N)

All scenarios were computed using all the models, except for BALTSEM which only ran the *Reference* and the *Manure investments* scenario, because the impacts on loads to the Baltic turned out to be small in *No greening*. Since the models generate large amounts of results, we focus on the results of the scenario *Manure investments*, which includes both removal of Greening and the introduction of all the technological changes, and use the results of *No greening* in selected places, such as in Table 4 and Fig. 2 to decompose and put the results of *Manure investments* in a context.

### Results from CAPRI on nutrient balances

Figure 2 shows manure nitrogen efficiency in each scenario following the computations in CAPRI. The results, which are in a similar range as those reported in Webb et al. (2013), indicate that manure management techniques can increase manure N efficiency in all countries but Denmark, where relevant technologies are already applied. The *Manure investments* scenario implies a catching up to 54% N efficiency, from diverse starting points. Therefore, fertilizer values differ across regions in *Reference* and *No greening*, but equalize in *Manure investments* by assumption. Removing only the greening requirements assumes no change in technology and does not affect manure efficiency, albeit surplus and leakage *are* affected through changed production activities.

Table 3 shows the impacts on the N budgets at the field level, computed by CAPRI for NUTS2 regions and aggregated to member states. All numbers are from the *Manure Investment* scenario which includes also the removal of greening, in total and as difference to the *Reference* scenario. The results are shown at national level for the EU member states contributing to the Baltic Sea drainage area, computed per total area and not only for the drainage basins of the Baltic Sea. Impacts per drainage basin are shown in the following section.

**Table 3:** Summary of nitrogen budgets at the field (outputs of CAPRI) in the *Manure investment* scenario, aggregated from NUTS2 to countries

Source (+) or sink (−)	Denmark	Germany	Finland	Sweden	Estonia	Lithuania	Latvia	Poland
+ Mineral fertilizers	160	1449	120	134	44	184	57	1125
	(− 6)	(− 243)	(− 21)	(− 41)	(− 7)	(− 38)	(− 37)	(− 175)
+ Manure^a^	250	1076	73	118	21	63	36	446
	(0)	(1)	(0)	(0)	(0)	(1)	(1)	(1)
+ Crop residues	151	1460	107	227	60	201	174	723
	(− 1)	(− 18)	(1)	(− 3)	(1)	(6)	(17)	(− 7)
+ N fixation	37	175	7	36	12	48	28	76
	(0)	(− 7)	(0)	(0)	(0)	(2)	(6)	(− 1)
+ Atm. deposition	48	177	13	33	9	29	20	162
	(− 1)	(− 4)	(− 1)	(− 1)	(0)	(0)	(0)	(− 5)
− Uptake by crops^b^	428	3401	239	406	95	382	253	1731
	(− 2)	(− 23)	(− 2)	(− 10)	(0)	(5)	(10)	(10)
= Total Surplus	218	936	80	143	52	143	63	801
	(− 7)	(− 248)	(− 18)	(− 36)	(− 6)	(− 33)	(− 23)	(− 197)

All values are in 1000 tonnes N, numbers in brackets are differences to Reference

^a^Net of losses in handling and storage

^b^“Uptake by crops” contains nutrients that become “Crop residues” on the source side of the computation. This way of defining crop residues is convenient because rotation of crops cause crop residues from one crop to benefit another

*Source* Own computations and simulations with CAPRI

Changes relative to *Reference* are due both to the change in technology and to changes in cropping patterns and animal herds that are induced by the changes in policy and crop nutrient availability/value. Included in the table are the inputs of nutrients from mineral fertilizer, manure, crop residues and atmospheric deposition as well as biological fixation. Uptake by crops removes nutrients from the soil, some of which is returned in the form of crop residues or manure. The total surplus is net of losses in storage and during application (ammonia). The nitrogen inputs at field level computed in CAPRI are inputs to the agro-hydrological model.

As manure efficiency increases, there is a reduction in N surplus from agriculture in all countries. When the nutrients in manure become more available to the plants, farmers need to supply less nutrients in the form of mineral fertilizers and we observe a decrease in the use of mineral fertilizers in all countries.

The largest impacts on N surplus are found in Germany and Poland, where total N surpluses are reduced by 248 000 and 197 000 tonnes per year, respectively. The impact in Germany is large because Germany is a large country. Given that Poland increases the FV of manure most of all countries (from 0.36 to 0.54, see Fig.  2), the reduction of total surplus found in Poland is surprisingly small—around 20% reduction of total surplus. That is of the same relative size as the impact in Sweden, where the gain in FV is much smaller (from 0.42 to 0.54, see Fig. 2). The explanation is that in Poland, and similarly for Lithuania, a smaller share of total N comes from manure, because agriculture is relatively more focussed on arable crops than on animals. Where less manure is handled, the impacts of improved manure technologies is smaller too.

The small increase of the utilization of manure as fertilizer happens because the higher FV makes manure more valuable to farmers. However, manure being only a minor part of the economic output from animals, the impacts on herd sizes and manure production are also minor. Deposition and N-fixation change slightly as a result of changes in the production mix and crop areas.

Table 4 analyses the impacts on total N-surplus and N-surplus per ha. In line with results of previous studies (e.g. Gocht et al. 2017), abolishing the greening decreases the total surplus (25 thousand tonnes, or about 1%). However, without the greening, there is also a reduction in the utilized agricultural area (UAA) of close to a million hectares, implying a more intensive agriculture with higher surplus per hectare, increased from 63.4 to 64.5 kg/ha. Depending on the location (riverine, proximity to watersheds etc.), intensification in nitrogen use can result in locally higher nitrogen surpluses and negative consequences for eutrophication.

**Table 4:** Decomposition of impacts on UAA and N-surplus, total for the eight Baltic countries, and for all three scenarios

	Agricultural area	Total *N* surplus	Surplus per ha
	(1000 ha)	(1000 t)	(kg/ha)
Reference	47,426	3005	63.4
No greening	46,244	2981	64.5
diff. to *Reference*	(− 1182)	(− 25)	(1.1)
All improvements	46373	2438	52.6
diff. to *No greening*	(129)	(− 543)	(− 11.9)
Partial technology implementations, diff. to *No greening*			
Only improved storage	(30)	(− 137)	(− 3.0)
Only improved application	(44)	(− 197)	(− 4.3)
Only liquid system	(19)	(− 60)	(− 1.3)
Improved storage and application	(69)	(− 319)	(− 7.0)
Improved storage and liquid system	(56)	(− 229)	(− 5.0)
Improved application and liquid system	(101)	(− 407)	(− 8.9)

Numbers in brackets are differences to Reference and No greening as indicated

*Source* own computations

Applying the new set of manure technologies in the *Manure investments* scenario dominates the effect of removing greening. In Table 4, we compute both absolute numbers and the difference to *No greening* to isolate the impact of only the technology change. We find that the improved technology could lead to an additional reduction of 543 thousand tonnes of N surplus annually. Some (129 thousand hectares) of the land abandonment in *No greening* is also reversed,^3^ so that in the bottom line, the N surplus per hectare is reduced by 11.9 kg/ha on average compared to the *No greening* scenario.

The bottom six lines of Table 4 how each technological measure and combinations of them influence the results. The “improved storage” measure is approximately additive with the other measures, and accounts for about ¼ of the total effect on surplus reduction. The measures “improved application” and “liquid system” are complementary. Increasing the rate of injection technology without any other measure reduces N surplus by 197 000 tonnes, and implementing “liquid system” alone only brings surplus down by 60 000 tonnes. However, combining those measures gives a reduction of 407 000 tonnes, which is considerably more than the sum of the impacts of the individual measures.

### Effects of improved manure management on N leaching and N loading to the Baltic Sea

**Fig. 3:**
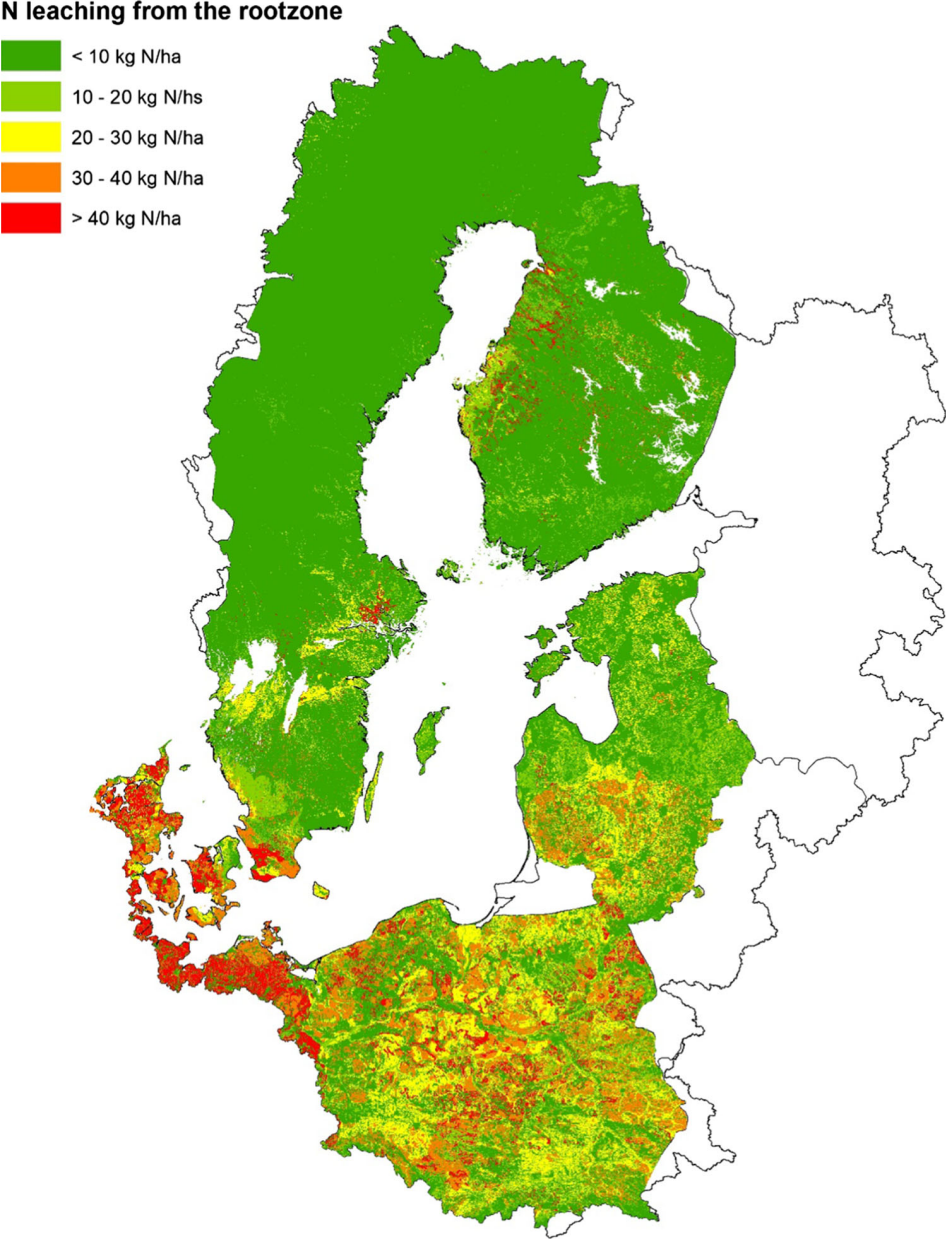
Nitrogen leaching from the rootzone for each HSMU, all land uses combined, *Reference* scenario. The map also shows the non-EU countries in the Baltic Sea drainage basin

Nitrogen leaching from the rootzone from both agricultural areas and from other land uses was calculated with the agro-hydrological nutrient transport model for all three scenarios. Nitrogen leaching at the HSMU level in the *Reference* scenario is shown in Fig. 3. Nitrogen losses are high in large parts of Denmark, Germany, the southernmost part of Sweden, and to some extent in Poland. Nitrogen losses are lower in the Baltic states, mid- and northern Sweden, and Finland. The northern part of the drainage basin is in a near-pristine state (Humborg et al. 2003).

**Fig. 4:**
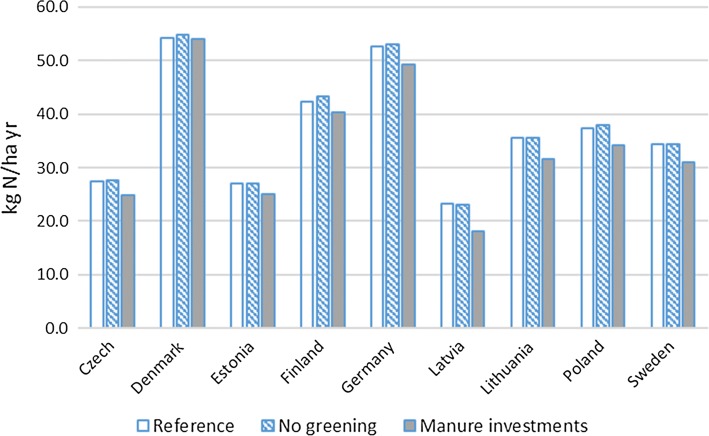
Nitrogen leaching in the scenarios (kg N/ha/year)

Nitrogen leaching from agriculture aggregated to the national level for the EU member states in the Baltic Sea drainage area is shown in Fig. 4 for all scenarios. Agricultural N losses are especially high in Denmark and Germany. The effect of introducing the Manure Investment scenario ranges from close to zero in Denmark to 22% in Latvia. The effect in Denmark is small because the scenario aims at extrapolating the present manure management in Denmark to the remaining countries. The reduced N losses in the scenario are a result of substituting mineral fertilizer with manure due to increased fertilizer value of manure and thus reducing the total N input. Consequently, larger effects will be seen in countries where (i) manure makes up a substantial part of total N input, and (ii) where the present manure storage and handling might be improved.

**Fig. 5:**
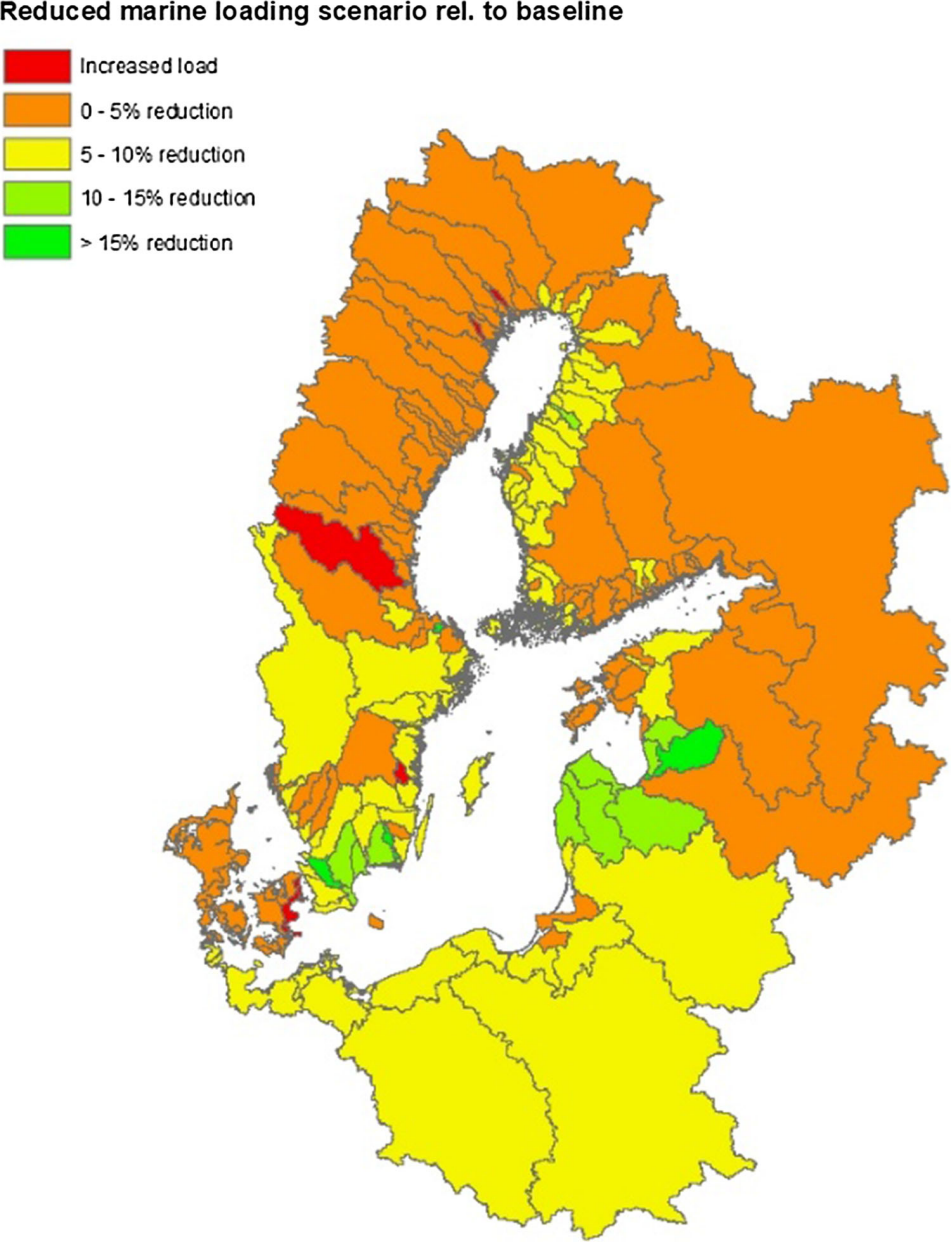
Relative change in N loading to the Baltic Sea for the *Manure investments* scenario relative to the reference. Data aggregated to 117 sub-basins

Nitrogen loading to the Baltic Sea was calculated by combining rootzone N losses with catchment based estimates of N retention (i.e. removal of nitrate due to denitrification) during transport in groundwater and surface waters. Nitrogen loading was calculated for 117 sub-basins and serves as inputs to the BALTSEM model. Figure 5 shows the relative change in loading resulting from the Manure Investment scenario relative to the reference. In total, the loading was reduced by 38 900 tonnes N or 7.4%

### Impacts on the Baltic Sea

**Fig. 6:**
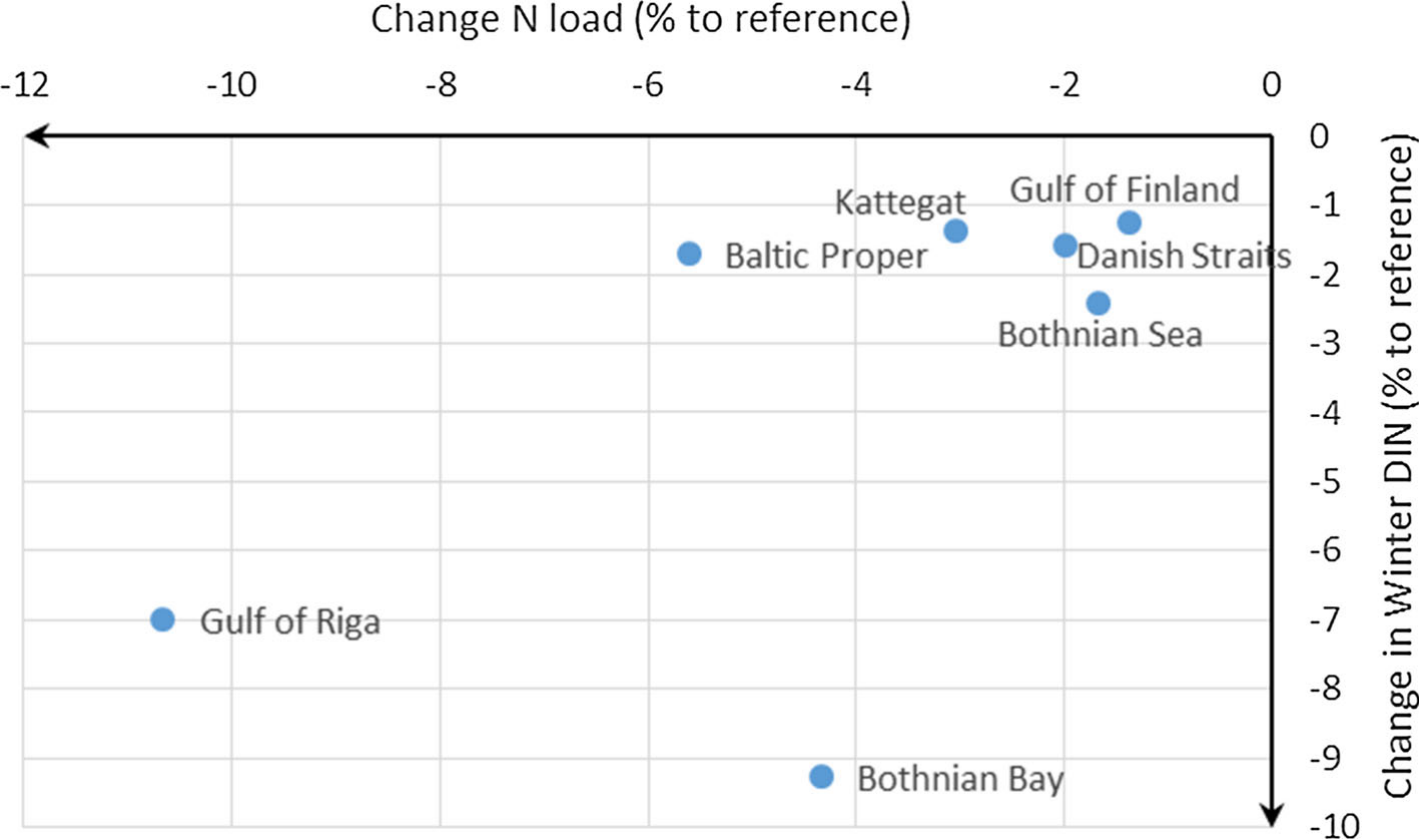
Changes between *Reference* and *Manure investments* scenarios (%) of total N loads per basin versus the resulting changes winter DIN concentration in the Baltic Sea basins

The change in loads to the Baltic Sea were imputed as perpetual annual changes following the *Manure investments* scenario vs a baseline scenario in the BALTSEM model. The total N load also contains point sources of N as well as atmospheric deposition, whence the relative reductions in N loads are less than implied by the changes in loads from agriculture. In addition, the ecosystem response in the Baltic Sea is strongly dependent on both nitrogen and phosphorus, with a predominant domination of nitrogen limitation of the phytoplankton production during spring and phosphorus limitation during summer. In this paper, we had to limit ourselves to quantify the N loading in the various scenarios, although one could anticipate some changes also to P loading. Therefore, it is not justifiable to investigate the implications on the environmental status in terms of indicators like plankton production and bloom intensities. Instead, we instead use BALTSEM to investigate the anticipated changes in winter inorganic nitrogen concentrations (DIN, representing the nitrogen readily available for primary production) that do provide a trustworthy scaling of the effects on the environment from the *Manure investments* scenario. Figure 6 shows the changes in total N loads for each of seven basins of the Baltic, and the resulting impact on DIN concentration.

The response of DIN concentrations to N loads is the result of complex internal nutrient cycling, comprising of both internal removal due to denitrification, as well as, at least in some sub-basins, fixation of atmospheric nitrogen by cyanobacteria. The largest relative reduction in N load, − 10.66%, happens in the Gulf of Riga (bottom left dot in the figure), and implies a reduction in N concentration of 7%. However, the largest reduction in N concentration, − 9.26%, happens in the northern part of the Baltic Sea, called the Bothnian Bay, despite a fairly small reduction in N load. Primary production in Bothnian Bay is strongly limited by phosphate implying that the loss of nitrogen through denitrification and sediment burial is not reduced when N loads decrease and therefore water column concentrations drop significantly. The large Baltic Proper contrast this by showing only a small reduction in N concentration, because here nitrogen fixing cyanobacteria counteract the load reduction, and in addition, reduced primary production in spring reduce somewhat the hypoxia in the basin which reduce somewhat denitrification. The Gulf of Finland is dominated by the loads from Russia, which are not included in this study and thus held constant. In the Danish Straits and Kattegat the main loads come from Denmark and Sweden, and since the impact on Danish agriculture was small, the impacts on those basins are also small.

## Conclusions

Technological measures for manure handling and application of manure have potential to reduce nitrogen surplus from agriculture more than the existing greening measures. Although the analysed changes of manure handling technologies provide substantial relative reductions of surpluses in agriculture, they translate to more moderate reductions in N loads to the Baltic Sea, of about 7% for the entire drainage basin, with significant variation between catchments. The general impact on nitrogen concentrations in the Baltic is smaller still, due to the constant loads of N from other sources and the dynamics of the sea. Therefore, the proposed measures can contribute to but are not sufficient to obtain a substantial improvement of the status of the Baltic Sea.

Decomposition of the impacts of increased storage, improved application technology and increased share of liquid manure reveals that the effects interact in both additive and complementary ways. Increased storage capacity improves timing of application and is beneficial almost regardless of application technology. In contrast, the more efficient technologies “injection” and “hoses” are only available for liquid manure, whereas solid manure only can be spread using broad-spread technology. Therefore, only investing in spreading technology is pointless if the share of liquid manure is very low, as is the case in Poland and Latvia. Efficient policies should take this complementarity into account, by stimulating modernization of farms towards liquid systems before addressing investments in spreading technology. According to our dataset, storage capacity (share of manure stored in facilities with at least nine months of capacity) is good in all countries except Poland (30%) and Lithuania and Latvia (60%), whereas there are high shares of inefficient broad-spread technologies in all countries except for Denmark.

As our data are compiled from heterogeneous sources and partly very old, updated data on farm technology are needed in order to properly identify lagging regions. Data on average manure storage capacity and spreading technologies are not available from Eurostat or farm survey data. For Sweden and Denmark, we could use national statistics containing values for average storage capacity; for the technology shares, we obtained data for Sweden, Denmark, Poland, Estonia and Finland. For the other countries, we used data from previous studies. Based on Eqs. 1 and 2, we were able to estimate the fertilizer value based on storage capacity and spreading technologies, which is essential in assessing the losses of nitrogen from agriculture. In the future, obtaining updated data on storage capacity from the other Baltic countries would improve this kind of estimates. In combination with data on investment costs, such data could be used in our set-up to compute the most cost-efficient measures for reducing N surplus.

In order to achieve further reductions of nutrient loads from agriculture, measures that reduce fertilization rates to a point slightly below the economic optimum would make sense, both from an empirical and from a theoretical point of view. From the empirical side, there is evidence from Denmark that such an approach can be made to work, albeit there is administrative overhead involved (Blicher-Mathiesen et al. 2014; Dalgaard et al. 2014). From a theoretical viewpoint, the economically optimal fertilization rates would in general not consider the public costs of nutrient leaching, and so be too high from an economic efficiency point of view. Economic instruments, such as taxes or trade mechanisms, can be used to internalize these costs. This recommendation is supported by the findings and that the nutrient load reductions are larger in areas with low nitrogen utilization and high animal density.

In the set-up of the scenarios, we assume that the budget currently spent on greening (about 12 billion euro annually for the EU as a whole) would be sufficient to cover the costs of the technological measures proposed here. However, we have not provided any evidence that that would actually be the case. Experiences from Denmark, where similar measures have been compulsory without any economic compensation for three decades, support the idea that the costs for agriculture would be manageable.

The analysis did not consider changes in phosphorous loads. Since phosphorous and nitrogen interact, an extension of the analysis to include phosphorous would be relevant. Such an extension is foreseen for a subsequent study.

## Electronic supplementary material

Below is the link to the electronic supplementary material.

Supplementary material 1 (PDF 294 kb)

## Footnotes

1 Except Croatia, which was not yet an EU member.

2 For detailed description of methodology see Bioteau et al. (2009).

3 Thanks to lower costs for crop nutrients and associated increases in profitability.
