# Supplementary material for: Can investments in manure technology reduce nutrient leakage to the Baltic Sea?
Source: Ambio. 2019 Oct 3;48(11):1264–77. doi: 10.1007/s13280-019-01251-5 (PMC6814651; doi:10.1007/s13280-019-01251-5)
Supplement: Supplementary file 1 — Supplementary material 1 (PDF 294 kb) [file 13280_2019_1251_MOESM1_ESM.pdf]

Ambio

Electronic Supplementary Material
This supplementary material has not been peer reviewed

Title: **Can investments in manure technology reduce nutrient leakage to the Baltic Sea?**

Torbjörn Jansson, Hans Estrup Andersen, Berit Hasler, Lisa Höglind, Bo G. Gustafsson

S1. Fertilization restrictions in CAPRI

We distinguish the three macro-nutrients N, P and K. The supply and uptake of those nutrients are modelled in a uniform way, save for the fact that there is fixation and atmospheric deposition only of N.

Each crop has a requirement per hectare, calculated based on the yield. Yields are exogenous from the vantage point of the producer, but there are alternative technologies available for each cropping activity, letting the producer choose between a higher input and higher yield technology and a lower input and lower yield technology. There is also a separable, i.e. handled outside of the optimization model, relation between prices and optimal yields.

From the basic nutrient requirement we first deduct the rate of biological fixation (only for nitrogen and selected crops). The remainder is inflated by a (calibrated) factor and additive term of over-fertilization, and then scaled with a soil-specific factor (only for nitrogen), to arrive at the total amount of nutrients that need to be supplied to the crop. This is the left hand side of Equation S1.1.

Nutrient supply, shown on the right hand side of Equation S1.1, comes from mineral fertilizer, manure, crop residues and atmospheric deposition. All three sources are subject to ammonia losses during application. For manure and crop residues, there are also availability factors applied to the nutrient content (denoted by $\phi_{r,\text{excr},n}$ in Equation S1.1), corresponding to the Fertilizer Value (FV) of manure relative to mineral fertilizer. The availability factor is a key parameter of interest in the simulations that we carry out in this study. Crop residues can be re-distributed among crop groups for annual arable crops but not for grassland and permanent crops, where it stays with the crop that produced it.

Equation S1.1: Fertilization requirement function in CAPRI

$$\sum_{i \in I_j,k} \left[levl_{rik} \left(ret_{rni} (1 - biofix_{rni}) \lambda_{rnik}^{prop} + \lambda_{rni}^{const} \right) soil_{rn} yf_{rnik} \right]$$

$$= fmine_{rnj}(1 - loss_{rn}) + fexcr_{rnj} \phi_{r,\text{excr},n} + fcres_{rnj}(1 - loss_{rn}) \phi_{r,\text{cres},n}$$

$$\forall r, j, n$$

Indices in Equation S1.1:

r	region	
i	crop	
j	crop group	
k	technological	
	nutrient	(N, P, K)

Endogenous choice variables in Equation S1.1:

$levl_{rik}$	Number of hectares of each crop
i$fmine_{rnj}$	Application of mineral fertilizer n to crop group j in region r
$fexcr_{rnj}$	Application of manure n to crop group j in region r
$fcres_{rnj}$	Allocation of crop residue n to crop group j in region r.

Parameters in Equation S1.1:

ret_{rni}	Uptake of nutrients by the crop
$biofix_{rni}$	Biological fixation, share (only for N and selected crops)
λ_{rnik}^{prop}	Over-fertilization factor, calibrated
λ_{rni}^{const}	Over-fertilization term, calibrated
$soil_{rn}$	Soil factor
yf_{rnik}	Yield factor for technologies
$loss_{rn}$	Loss rate
$\phi_{r,\text{excr},n}$	Nutrient availability factor for manure
$\phi_{r,\text{cres},n}$	Nutrient availability factor for crop residues

The reader may have noted that there is no loss rate for manure in Equation S1.1. CAPRI does contain such loss rates, but they are specific for each animal type and therefore happens on the manure supply side of the regional manure balance (see Equation S1.2).

The model contains three types of manure: N-manure, P-manure and K-manure. From an agricultural point of view this may seem odd. It might be more intuitive to think of one type of manure per animal category. The motivation is to keep the system simple and flexible. With the present representation, where each animal category supplies N, P, and K-manure, the number of manure classes can be limited and yet the unique mix of nutrients from each animal category can be defined.

The supply of each manure type is collected in a "pool" for each regional farm model, i.e. for each NUTS2-region. Regions within a member state may trade manure, subject to a cost. The supply in the pool plus the traded quantities has to be distributed to the crops in the region, i.e. there is an equality-restriction in place.

Equation S1.2. Regional manure nutrient balance

$$\sum_j fexcr_{rnj} + \sum_s T_{rs} nutshr_{rn} = \sum_{ik} levl_{rik} o_{rnik} (1 - loss_{rin})$$

Where

o_{rnik}	is the output of manure nutrient n from animal type i using technology k in region r,
$nutshr_{rn}$	is the average content of each nutrient in the regional manure pool,
T_{rs}	is the quantity of manure traded from r to s, and other symbols and indices as before

S2: Parameters and variables for the calculation of fertilizer value

Recall that we compute fertilizer value as in equation 1 in the main text, reprinted here for convenience. The parameters FV_{kit} are given in table 2 of the main text.

$$FV_r = \sum_{kit} FV_{\text{kit}} a_{rk} b_{rki} c_{rt}$$

Table S2.1. Parameters in the *Reference* scenario for estimation for the fertilizer value.

	a: Liquid share[a]	x: Share of storage capacity > 9 months[b]	b: technology share[c]			Fertilizer Value
			Broad spread	injection	hoses	
Denmark	0.78	0.90	0.009	0.463	0.528	0.53
Germany	0.67	0.60	0.542	0.198	0.260	0.43
Sweden	0.36	1.00	0.225	0.201	0.574	0.43
Finland	0.31	1.00	0.433	0.325	0.242	0.42
Poland	0.18	0.30	0.935	0.023	0.042	0.36
Estonia	0.49	1.00	0.387	0.419	0.194	0.45
Lithuania	0.45	0.60	0.831	0.012	0.156	0.38
Latvia	0.15	0.60	0.800		0.200	0.37

[a]Data from CAPRI, originating from Velthof et al., 2009.

[b]This is used in equation 2 of the main text to compute the application share c_{rt} in each period *t* for each region *r*. The coefficients of the linear function are found in table S3.2

[c]Data from farm survey (Hasler et al. 2018) and Bioteau, 2009. See materials and methods. Only valid for liquid manure. For solid manure, the share of broad spread is assumed to be 100%.

Table S2.2. Parameters in the *Manure investment* scenario for estimation for the fertilizer value after investments that improve parameters a, *x* and b.

	a: Liquid share	x: Share of storage capacity > 9 months	b: technology share			Fertilizer Value
			Broad spread	injection	hoses	
Denmark	0.78	1.00	0	0.50	0.50	0.55
Germany	0.75	1.00	0	0.50	0.50	0.54
Sweden	0.75	1.00	0	0.50	0.50	0.54
Finland	0.75	1.00	0	0.50	0.50	0.54
Poland	0.75	1.00	0	0.50	0.50	0.54
Estonia	0.75	1.00	0	0.50	0.50	0.54
Lithuania	0.75	1.00	0	0.50	0.50	0.54
Latvia	0.75	1.00	0	0.50	0.50	0.54

S3: Estimates of fertilizer timing as a function of storage capacity

The timing of application of liquid manure as a function of storage capacity is found by linear regression (equation 2 in the main text):

$$c_{rt} = \beta_t x_r + \alpha_t$$

On the left hand side, $c_{r,t}$ shows the share of liquid manure that is applied in each of the three time periods $t = \{\text{spring,summer/fall,winter}\}$, in each region r, depending on the regional average storage capacity x_r and the two estimated parameters α_t and β_t. Storage capacity is defined as the share of liquid manure that is kept in storage facilitates with at least 9 months of capacity, as this is the minimum capacity allowing optimal timing of application of manure. The function is linear, and such that $\sum_t c_{rt} = 1$ for any x_r. The Durbin-Watson test demonstrated that there is no statistical evidence that the error terms in the regression models are auto-correlated, table S3.1. The resulting coefficients for equation 2 are shown in table S3.2, and their graphs printed in figure S3.1.

Table S3.1: Results of the Durbin-Watson test

		**p-value**
**Winter application**	Pr < DW	0.1958
	Pr > DW	0.8042
**Spring application**	Pr < DW	0.1699
	Pr > DW	0.8301
**Summer/autumn application**	Pr < DW	0.4119
	Pr > DW	0.5881

Note: Pr < DW is the p-value for testing positive autocorrelation, and Pr > DW is the p-value for testing negative autocorrelation.

Table S3.2. Parameters in equation 2, estimating timing of manure application as a function of storage capacity. R^2: coefficient of determination; n: number of observations.

	$\boldsymbol{\alpha_t}$	$\boldsymbol{\beta_t}$	**R^2**	**n**
**Spring application**	29.383	61.41	0.9724	8
**Winter application**	17.714	- 17.8	0.6201	8
**Summer/autumn application**	52.902	- 43.61	0.9587	8

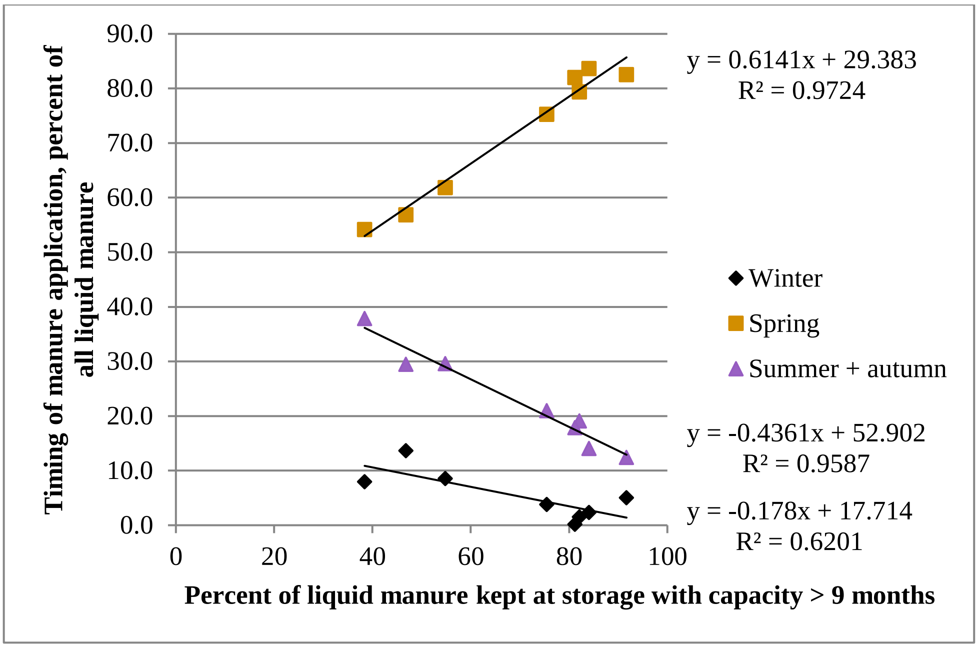

Figure S3.1: Estimated relation between timing of application and storage capacity in Denmark. Data from Grant et al. (1999).
